# High-Throughput Identification of Organic Compounds from Polygoni Multiflori Radix Praeparata (*Zhiheshouwu*) by UHPLC-Q-Exactive Orbitrap-MS

**DOI:** 10.3390/molecules26133977

**Published:** 2021-06-29

**Authors:** Shaoyun Wang, Xiaozhu Sun, Shuo An, Fang Sang, Yunli Zhao, Zhiguo Yu

**Affiliations:** School of Pharmacy, Shenyang Pharmaceutical University, Shenyang 110016, China; wsy17862968828@163.com (S.W.); sxz13591173561@163.com (X.S.); a13831181394@163.com (S.A.); sangfang03@163.com (F.S.)

**Keywords:** polygoni multiflori radix praeparata, UHPLC-Q-Exactive Orbitrap-MS, chemical constituents, traditional Chinese medicine

## Abstract

Polygoni Multiflori Radix Praeparata (PMRP), as the processed product of tuberous roots of *Polygonum multiflorum* Thunb., is one of the most famous traditional Chinese medicines, with a long history. However, in recent years, liver adverse reactions linked to PMRP have been frequently reported. Our work attempted to investigate the chemical constituents of PMRP for clinical research and safe medication. In this study, an effective and rapid method was established to separate and characterize the constituents in PMRP by combining ultra-high performance liquid chromatography with hybrid quadrupole-orbitrap mass spectrometry (UHPLC-Q-Exactive Orbitrap-MS). Based on the accurate mass measurements for molecular and characteristic fragment ions, a total of 103 compounds, including 24 anthraquinones, 21 stilbenes, 15 phenolic acids, 14 flavones, and 29 other compounds were identified or tentatively characterized. Forty-eight compounds were tentatively characterized from PMRP for the first time, and their fragmentation behaviors were summarized. There were 101 components in PMRP ethanol extract (PMRPE) and 91 components in PMRP water extract (PMRPW). Simultaneously, the peak areas of several potential xenobiotic components were compared in the detection, which showed that PMRPE has a higher content of anthraquinones and stilbenes. The obtained results can be used in pharmacological and toxicological research and provided useful information for further in vitro and in vivo studies.

## 1. Introduction

Polygoni Multiflori Radix Praeparata (Zhiheshouwu in Chinese, PMRP), as a processed root of *Polygonum multiflorum* Thunb. (heshouwu in Chinese, PMR), has a long history in clinical application. The common processing method of PMRP is steaming or boiling PMR with a black bean decoction, as prescribed by the Chinese Pharmacopoeia. After processing, the concentrations of major components and traditional usage have changed. PMR contains more combined anthraquinones, while fewer stilbenes and more free anthraquinones are found in PMRP [1]. PMRP could enhance immune function, nourish the liver and kidney, prevent premature loss of hair, protect the nervous system, and inhibit atherosclerosis et al. [2,3,4]. Modern research has revealed that anthraquinones, stilbenes, flavonoids, and phenolic acids in PMRP are the major compounds of its pharmacological activities [5,6]. Several polyhydroxy stilbenes such as 2,3,5,4′-tetrahydroxystilbene-2-*O-β*-d-glucoside(THSG) have a similar structure to resveratrol, and they have also been proven to have a strong ability to antioxidize and perform free radical scavenging activities [7]. Besides, THSG show great lipid-regulation and protection against neurotoxicity [8]. Anthraquinones are the major compounds with extensive activity, such as anti-tumor, antibacterial, and neuroprotective effects. Emodin induces neuronal differentiation through PI3K/Akt/GSK-3β pathways in Neuro2a cells [9]. Three anthraquinones, including physcion, emodin, and questin, were regarded as Cdc25B phosphatase inhibitors by strongly inhibiting the growth of human colon cancer cells [10]. Proanthocyanidins, isolated from MPRP, have the potential to be functional ingredients in reducing postprandial hyperglycemia, by inhibiting α-amylase and α-glucosidase [11].

However, with the widespread application of PMRP in the clinic, many adverse events of PMRP, including dyspnea, fever, rash, nephrotoxicity, and hepatotoxicity, have been reported in many countries such as Japan, China, Korea, Italy, Singapore, Spain, Australia, and the USA [12,13,14]. As the main organ of drug metabolism, the liver seems to be more susceptible to xenobiotic components. Therefore, the incidence of liver injury induced by PMRP has increased year by year [15]. Though some compounds of PMRP have positive physiological effects [16,17], there have been many studies reporting that several xenobiotic compounds could induce idiosyncratic hepatotoxicity. Anthraquinones are generally assigned as the major compounds of xenobiotics, because other anthraquinone-containing herbal medicines were also reported to induce liver injury [18,19]. Constituents other than anthraquinones, such as stilbenes and phenolic acids, were also considered to have a major contribution to the idiosyncratic hepatotoxicity of PMRP [20]. To find the potentially xenobiotic components and mechanisms of hepatotoxicity, qualitative and quantitative research has been explored. Zhang et al. [21] reported that the emodin-8-*O-β*-d-glucoside (EG) could induce hepatotoxicity, and the combination of EG and THSG could cause more severe liver injury. Moreover, in previous literature, the THSG, physcion and emodin showed no, moderate and severe cytotoxicity, respectively [22]. Rhein, which has weaker toxicity than emodin, has been demonstrated to exert concentration- and time-dependent toxic effects on L-02 cells [23].

At present, only a few compounds have been explored in xenobiotic studies. Considering the multi-component and multi-target characteristics of traditional Chinese medicine, the chemical constituents of PMRP should be identified for further studies. Meanwhile, previous studies have suggested that PMRP, extracted with different extraction solvents, showed various degrees of liver injury, and the order of toxicity was described as PMRP ethanol extract (PMRPE) > PMRP water extract (PMRPW) [24,25]. Consequently, to identify and compare the different components between PMRPE and PMRPW, an effective and sensitive ultra-high performance liquid chromatography coupled with hybrid quadrupole-orbitrap mass spectrometry (UHPLC-Q-Exactive Orbitrap-MS) method was established for characterization of the constituents of them. The results of this investigation are meaningful, and would provide a material basis for further pharmacological and toxicological studies.

## 2. Results and Discussion

### 2.1. Optimization of LC and MS Conditions

LC conditions including mobile phase, flow rate, column, and column temperature were optimized to obtain a good separation and resolution. Compared with methanol, using acetonitrile as the organic phase showed stronger elutive power and detection sensitivity. Due to most compounds in PMRP contain carboxyl and phenolic hydroxyl, the addition of 0.1% formic acid in the phase system can obtain better mass spectrometric responses and improve the shapes of most peaks. Therefore, the mobile phase was acetonitrile (A)-0.1% formic acid in water (B), with optimized gradient elution. The Waters HSS T3 column (2.1 mm × 100 mm, 1.8 μm, UK) is suitable for the high polar compounds and high percentage of the aqueous phase, which have been applied to the characterization of the constituents of other botanical extracts. For the MS conditions, we chose the negative mode by comparing the intensity of compounds in both positive and negative modes. Meanwhile, according to the base peak intensity chromatograms (BPC), more compounds can be detected in the negative mode. Finally, other MS parameters were optimized to obtain high sensitivity for most compounds. The results indicated that the UHPLC-Q-Exactive Orbitrap-MS developed in this study is appropriate to detect the chemical constituents in PMRP.

### 2.2. Identification of the Chemical Constituents in PMRP

**Figure 1:**
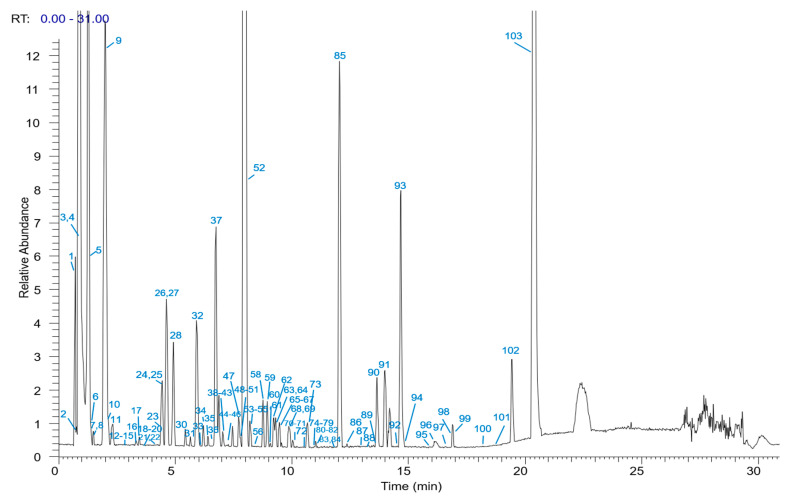
UHPLC-Q-Exactive Orbitrap-MS base peak intensity chromatogram (BPC) of PMRPE.

**Figure 2:**
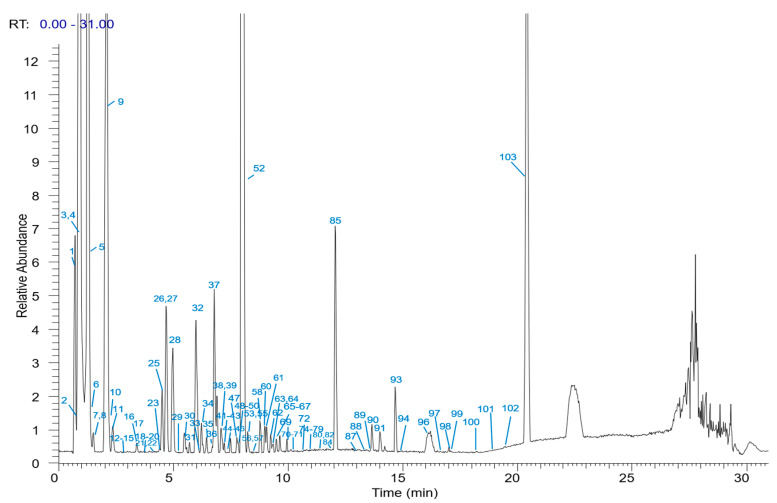
UHPLC-Q-Exactive Orbitrap-MS base peak intensity chromatogram (BPC) of PMRPW.

An in-house database that includes chemical names, molecular formulas, accurate molecular mass, chemical structures, and relevant fragments was established by searching Science Direct of Elsevier, Chemspider, PubMed, and CNKI (Chinese National Knowledge Infrastructure). We used Xcalibur™ and TraceFinder to obtain accurate mass, elemental composition, and multiple-stage mass data. By matching the in-house database to compare and characterize the compounds in PMRPE and PMRPW, these formulas which have been reported in the literature can be considered. A total of 103 chemical constituents were tentatively represented, including 24 anthraquinones, 21 stilbenes, 15 phenolic acids, 14 flavones, and 29 other compounds. The base peak intensity chromatogram (BPC) is shown in Figure 1 and Figure 2. The details of the identified compounds are summarized in Table 1 and the chemical structures of major constituents are shown in Figure S1.

#### 2.2.1. Identification of Anthraquinones and Derivatives

**Figure 3:**
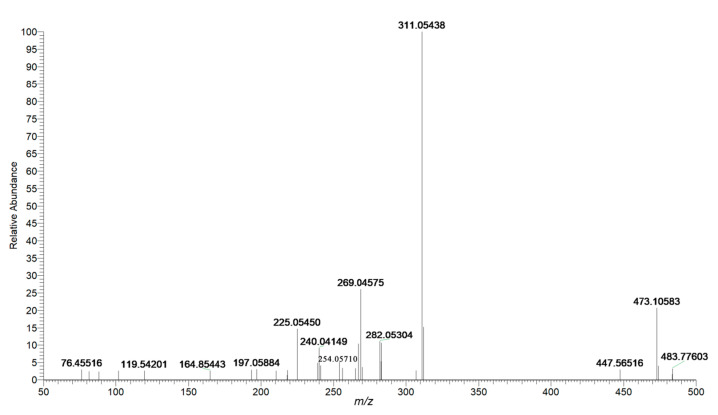
Mass spectrum of 2-acetylemodin-8-*O-β*-d-glucoside in negative mode.

**Figure 4:**
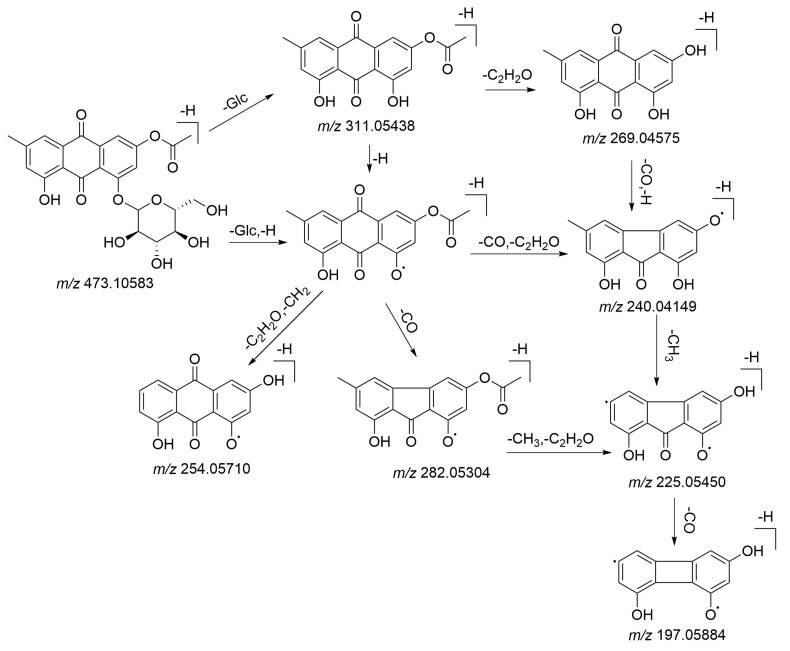
The proposed fragmentation pathways of 2-acetylemodin-8-*O-β*-d-glucoside.

Anthraquinones, which have the pharmacological effects of being anti-inflammatory, anti-virus, anti-cancer, lipid-lowering, and anti-diabetes [26], are the primary compounds in PMRP. There has been much literature which has revealed that anthraquinones can attenuate liver damage and demonstrate an anti-cirrhosis effect by reducing lipid peroxidation and inhibiting the proliferation of hepatic stellate cells [27,28,29]. Moreover, emodin and its oxidative metabolites were deemed as the main xenobiotic components, as they can combine with glutathione (GSH) to disturb cellular GSH and fatty acid metabolism in the liver [30,31]. Most anthraquinones in this family produced the characteristic fragment ions at *m*/*z* 269 and *m*/*z* 240, and the loss of two CO sequentially could be considered as the characteristic fragment behavior of anthraquinones and their derivates. In detail, peak **84**, with a retention time of 11.97 min, generated an [M–H]^−^ ion with mass accuracy at *m*/*z* 473.10583. The molecular formula was predicted as C_23_H_22_O_11_ using Xcalibur (Thermo Fisher Scientific) within 5 ppm. As shown in Figure 3, the characteristic fragment ions at *m*/*z* 311.05438 indicated a loss of glucuronic acid from the precursor ion at *m*/*z* 473.10583. Characteristic ions at *m*/*z* 269.04575 and 282.05304, which could be identified as losing C_2_H_2_O and CO from *m*/*z* 311.05438, respectively, were obtained. The [M−H]^−^ ion fragmented into other characteristic ions at *m*/*z* 254.05710, *m/z* 240.04149, and *m*/*z* 225.05450, which corresponded to [M-H-glc-C_2_H_2_O-CH_2_]^−^, [M-H-glc-C_2_H_3_O-CO]^−^ and [M-H-glc-C_2_H_3_O-CO-CH_3_]^−^. It was putatively identified as 2-acetylemodin-8-*O-β*-d-glucoside, and the proposed fragmentation pathways of 2-acetylemodin-8-*O-β*-d-glucoside are depicted in Figure 4. Peak **90** was found at 13.65 min and showed a precise molecular weight at *m*/*z* 283.06113. The fragment ion at *m*/*z* 268.03760 was produced by losing CH_3._ Other characteristic ions at *m*/*z* 240.04179 and 212.04668 were observed by losing two CO successively. According to the in-house database and reference standard, compound **90** was identified as physcion. Peak **93** was found at 14.69 min and generated a [M−H]^−^ ion at *m*/*z* 299.05493. MS/MS fragment at *m*/*z* 268.03696, 253.04982, and 240.04204 have corresponded to [M-H-CH_3_O]^−^, [M-H-CH_3_O-CH_3_]^−^ and [M-H-CH_3_O-CO]^−^. As a result, the compound was putatively identified as questinol. Peak **103** produced [M−H]^−^ ions at *m*/*z* 269.04514, and further characteristic fragment ions were acquired at *m*/*z* 241.04941 and 213.05467 by losing two CO successively. By comparing with the reference standard, the compound was identified as emodin.

#### 2.2.2. Identification of Stilbenes and Derivatives

**Figure 5:**
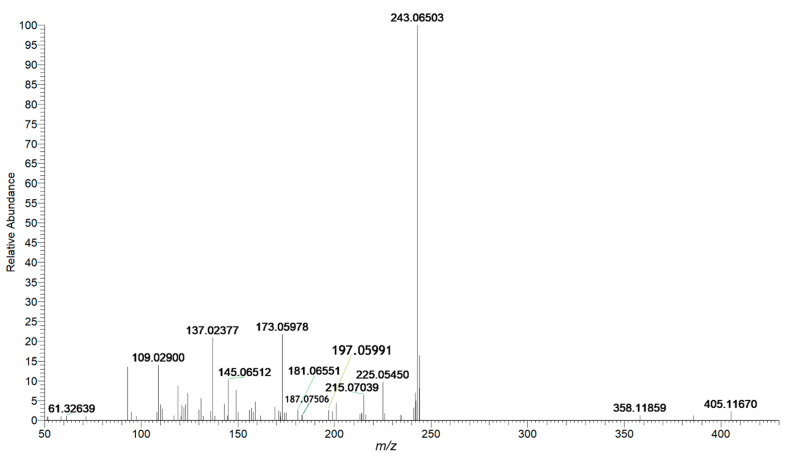
Mass spectrum of THSG in negative mode.

**Figure 6:**
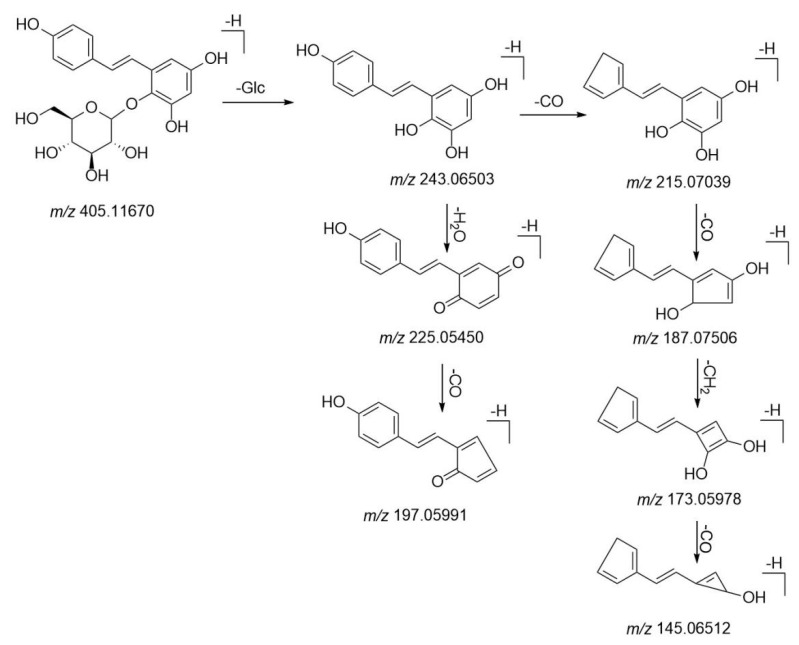
The proposed fragmentation pathways of THSG.

Stilbenes are the main characteristic components in Polygoni Multiflori Radix Praeparata, showing great lipid-regulating and antioxidant activity [8]. Specifically, THSG as a unique active constituent plays a vital role in hepatoprotective effects, with various abilities as to the improvement of mitochondrial function and the clearance of intracellular reactive oxygen species [32,33]. On the other hand, some studies have reported that THSG was regarded as a contributor to liver injury associated with the transformation of trans-THSG to cis-THSG [34]. Stilbenes and its derivatives displayed characteristic fragment ions at *m*/*z* 405 and *m*/*z* 243 in negative ion mode. The other two prominent ions at *m*/*z* 225 and *m*/*z* 215 were obtained as loss CO and H_2_O in A-ring after rearrangement, respectively. In detail, peak **52** was found at 7.94 min and generated an [M–H]^−^ ion at *m*/*z* 405.11670. The characteristic ion at *m*/*z* 243.06503 was produced by losing C_6_H_10_O_5_ from the precursor ion. Other characteristic ions at *m*/*z* 225.05450 and 215.07039 were obtained by losing H_2_O and CO from *m*/*z* 243.06503, respectively. Compound **52** was identified as THSG by comparing the reference standard. Figure 5 shows the MS/MS mass spectrum of THSG. The details of proposed fragmentation pathways are depicted in Figure 6. Peaks **67** and **69** were observed at 9.57min and 9.95min, respectively. Their molecular formulas were predicted as C_27_H_26_O_13_ within 5 ppm. They all produced fragment ions at *m*/*z* 405.11, 243.06 and 225.05, which were indicated as [M–H–gal]^−^, [M–H–gal–glc]^−^, [M–H-gal–glc–H_2_O]^−^, respectively. Although it was difficult to distinguish them by MS spectra, it was easier to identify them by comparing their retention time. According to the in-house database, the two compounds were Tetrahydroxy-stilbene-*O*-(galloyl)-glucoside and Piceatannol-3-*O-β*-d-(6″-*O*-galloyl)-glucoside. Based on the different positions of hydroxy in the benzene ring, the dehydration ability of them was different. Tetrahydroxystilbene-*O*-(galloyl)-Glucoside is more polar and can be more quickly eluted than Piceatannol-3-*O-β*-d-(6″-*O*-galloyl)-glucoside on reserved phase column. Therefore, peak **67** was putatively identified as Tetrahydroxystilbene-*O*-(galloyl)-Glucoside, and peak **69** was Piceatannol-3-O-β-d-(6″-*O*-galloyl)-glucoside. Peak **68** was found at 9.87 min and generated [M–H]^−^ ion at *m*/*z* 447.12930. MS/MS fragment at *m*/*z* 243.06511 and 225.05455 corresponded to [M–H–glc–acetyl]^−^ and [M–H–glc–acetyl–H_2_O]^−^. Compound **68** was putatively identified as 2,3,5,4’-tetrahydroxy-stilbene-2-O-(2”-*O*-acetyl)-*β*-d-glucoside.

#### 2.2.3. Identification of Flavonoids and Derivatives

**Figure 7:**
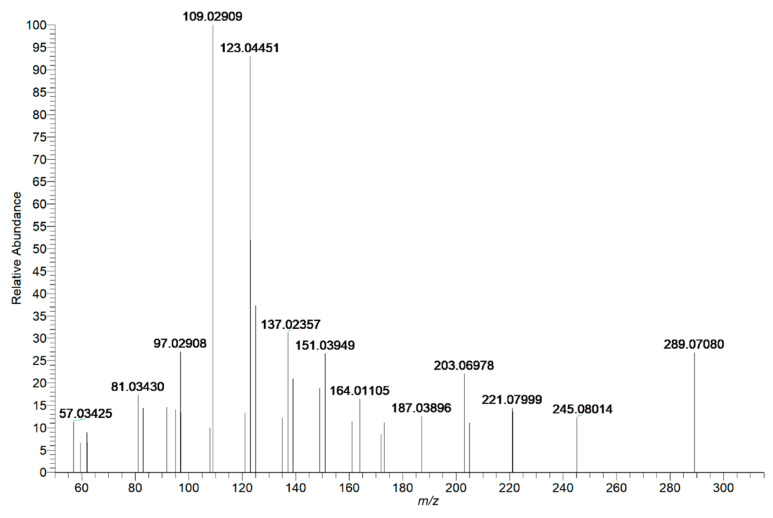
Mass spectrum of epicatechin in negative mode.

**Figure 8:**
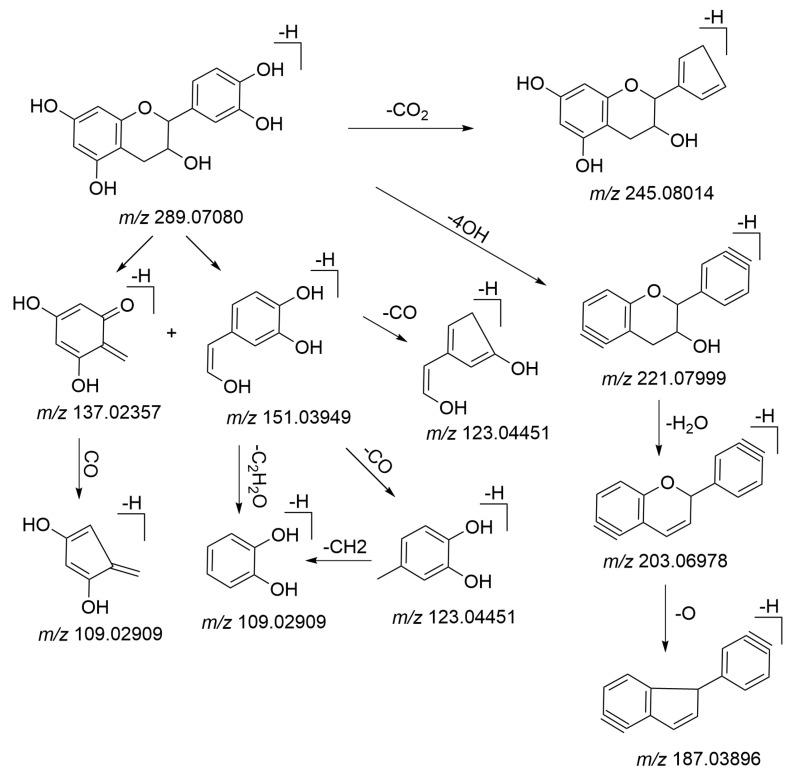
The proposed fragmentation pathways of epicatechin.

As the main antioxidant in the root, flavonoids and their derivatives exhibit antioxidant and free radical scavenging activities [35]. In addition, flavonoids can protect against liver injury through the regulation of NF-κB/IκBα, p38 MAPK, and Bcl-2/Bax signaling [36]. There were 14 compounds tentatively identified as flavonoids and their derivatives. Catechin and epicatechin are isomers and they were used as examples to illustrate the characterization process of flavonoids, which can undergo an RDA reaction by cleavage of C3–C4 and C2–C1 bonds of the C ring rearranging, and produced the characteristic fragment ions of *m*/*z* 151 and *m*/*z* 137. Figure 7 shows the MS/MS mass spectrum of epicatechin. The proposed fragmentation pathways are depicted in Figure 8. Peak **28** and Peak **31** generated an [M–H]^−^ ion at *m*/*z* 289.07111 and 289.07080, respectively. Their molecular formulas were all predicted as C_15_H_14_O_6_ within 5 ppm. The common characteristic ions were observed at *m/z* 151.03, 37.02, 123.04. The precursor ions undergo an RDA reaction to produce *m*/*z* 151.03 and 137.02, corresponding to [M-H-C_7_H_5_O_3_]^−^ and [M-H-C_8_H_7_O_3_]^−^, respectively. Characteristic ions at *m*/*z* 123.04 were obtained by losing CO from *m*/*z* 311.05438. Based on their different hydroxyl configuration at the C3 position and combined with the literature, catechin is more polar than epicatechin. Therefore, in this separation condition, the retention time of catechin is shorter. Peak **28** was putatively identified as catechin. Peak **31** was epicatechin.

#### 2.2.4. Identification of Phenolic Acids and Derivatives

**Figure 9:**
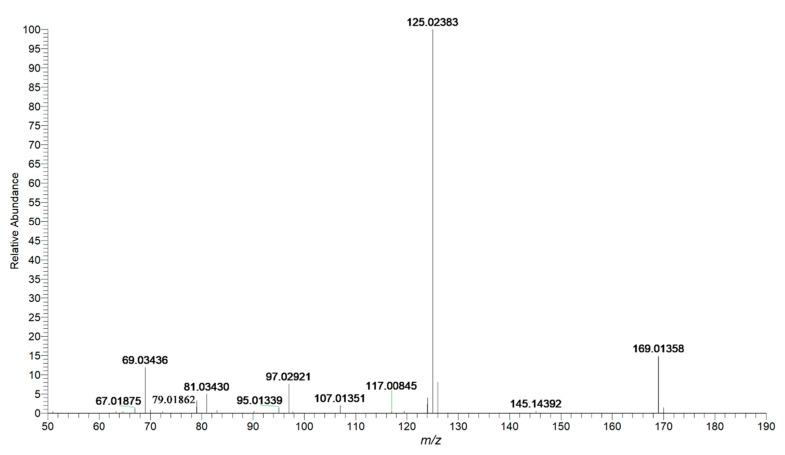
Mass spectrum of gallic acid in negative mode.

**Figure 10:**
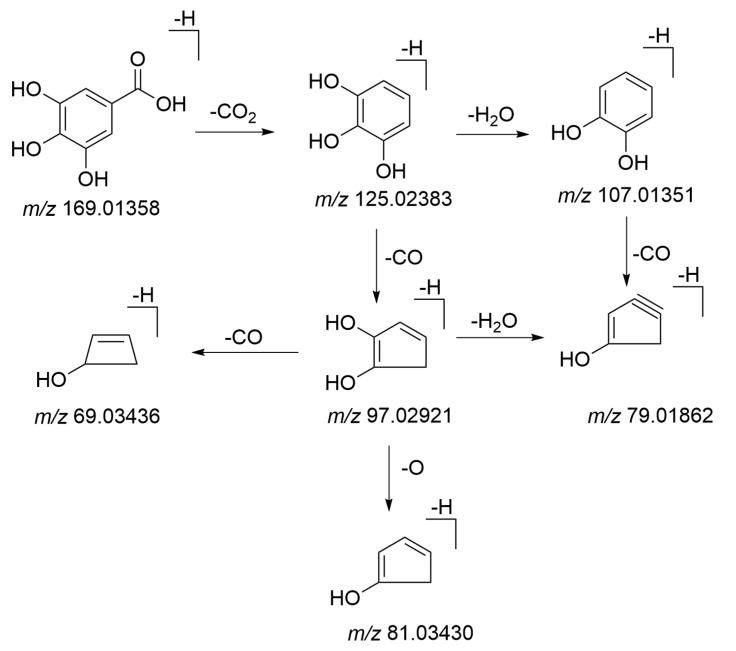
The proposed fragmentation pathways of gallic acid.

Fifteen phenolic acids and their derivatives were tentatively identified in PMRP. It has been reported to exhibit hepatoprotective effects and good inhibitory activity towards α-glucosidase[37]. Phenolic acids were structures containing one or more phenolic hydroxyl moieties. Therefore, the loss of 44 Da (−COO) and 18Da (H_2_O) could be considered as the characteristic fragment behavior of phenolic acids and its derivatives. Gallic acid was reported as a potentially xenobiotic component with anti-inflammatory activities and hepatotoxicity[38,39]. As shown in Figure 9, peak 9 with a retention time of 1.99 min generated an [M–H]^−^ ion with mass accuracy at *m*/*z* 169.01358. The characteristic ion at *m*/*z* 125.02383 was produced by losing CO_2_ from the precursor ion. The consecutive loss of CO_2_ and H_2_O leads to characteristic ions at *m*/*z* 125.02383 and 107.01351. Compound **9** was identified as gallic acid by comparing with the reference standard. The proposed fragmentation pathways of gallic acid are illustrated in Figure 10. Peak **17** was found at 3.34 min and generated an [M–H]^−^ ion at *m*/*z* 315.06995. It produced fragment ion at *m*/*z* 153.01865 by losing 162 Da which be considered as a loss of C_6_H_10_O_5_ group, and ion at *m*/*z* 109.02901 by losing a CO_2_ (44 Da). Therefore, compound **17** could be tentatively identified as protocatechuic acid-*O*-glucoside.

#### 2.2.5. Identification of Other Compounds

Peak **13** generated an [M−H]^−^ ion at *m*/*z* 125.02386, and the molecular formula was predicted as C_6_H_6_O_3_ within 5 ppm. Diagnostic ions at *m*/*z* 97.02918 and 81.03435 indicated the loss of CO and CO_2_ groups, respectively. Peak **13** was putatively identified as 5-Hydroxymethylfurfural, matched with the in-house database. Peak **22** was found at 4.35 min and produced an [M–H]^−^ ion at *m/z* 189.05476. The diagnostic fragment ions such as *m*/*z* 174.03186 and 161.06018 corresponded to [M–H–CH_3_]^−^ and [M–H–CO]^−^. Compound **22** was tentatively identified as Altechromone A. The [M−H]^−^ ion at *m*/*z* 151.03902, which was found at 6.54 min in peak **36**, indicated a molecular formula of C_8_H_8_O_3_ within 5 ppm. It produced characteristic fragments at *m*/*z* 136.01607, 123.04456, and 107.04993 due to the elimination of [M–H–CH_3_]^−^, [M–H–CO]^−^ and [M–H–CO_2_]^−^, respectively. Finally, peak **36** was tentatively identified as Vanillin. Aside from the major compounds analyzed above, the remaining constituents were also identified by comparing them with the in-house database.

#### 2.2.6. Comparison of Chemical Constituents Between PMRPE and PMRPW

Based on the identification strategy we have established, 101 components were identified in PMRPE and 91 components were identified in PMRPW. The results showed that there were 12 characteristic components in PMRPE, which were 2-vinyl-1H-indole-3-carboxylic acid, syringic acid, epicatechin-O-gallate, 2-methyl-5-carboxymethyl-7-hydroxychromone, epimedium, 2,3,5,4’-tetrahydroxystilbene-2-*O*-(2”-*O*-acetyl)-β-d-glucoside, 2,3,5,4’-tetrahydroxystilbene-2-*O*-β-d-(2″-*O*-coumaroyl)-glucoside, tetrahydroxystilbene-*O*-(caffeoyl)-glucoside, torachrysone, emodin-*O*-glucoside-gallate, chrysophanol anthrone and digitolutein. Two characteristic constituents were identified in PMRPW, which were polygonumosides C and di-emodin-di-glucoside.

**Figure 11:**
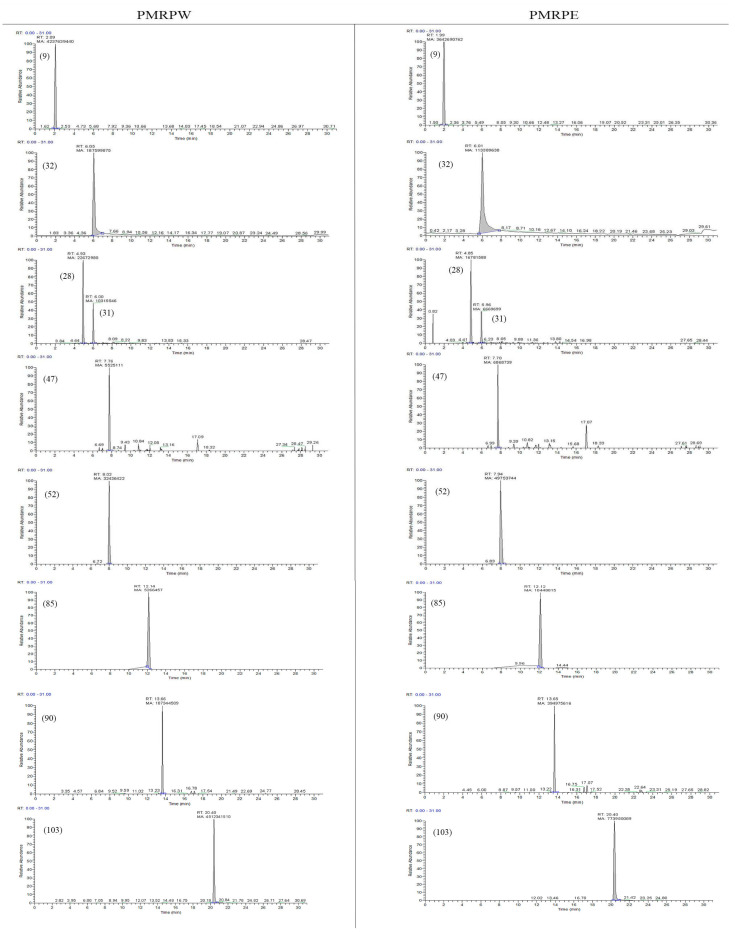
The chromatograms of potentially xenobiotic compounds in PMRPW and PMRPE.

Meanwhile, the peak area of potentially xenobiotic compounds reported in previous studies has been compared in PMRPW and PMRPE [1,40,41]. The representative chromatograms of potentially xenobiotic compounds are shown in Figure 11, and the specific parameters are listed in Table 2.

## 3. Materials and Methods

### 3.1. Materials and Chemicals

HPLC grade acetonitrile (ACN) and acetic acid were purchased from Sigma-Aldrich (St. Louis, MO, USA), and Dikma Technology Co., Ltd. (Beijing, China). Ethanol (industrial grade) was obtained from Shandong Yuwang Industry Co., Ltd. (Shandong, China). Purified water was obtained from the Hangzhou Wahaha Corporation (Hangzhou, China). PMRP was bought from GuoDa Pharmacy (Shenyang, Liaoning, China) and was identified as the processed root of *Polygonum multiflorum* Thunb. by professor Zhiguo Yu of the Shenyang Pharmaceutical University. The reference standards of emodin, physcion, and gallic acid were purchased from Chengdu MUST Bio-Technology Co., Ltd. (Sichuan, China). Chrysophanol, rhein and polydatin were obtained from the National Institute for the Control of Pharmaceutical and Biological Products (NICPBP, Beijing, China). Emodin-8-*O-β*-d-glucoside and 2,3,5,4-tetrahydroxystilbene-2-*O-β*-d-glucoside (THSG) were obtained from Sichuan Victory Bio-Technology Co., Ltd. (Sichuan, China). The purity of all the reference substances was higher than 98%.

### 3.2. Standard Solutions and Sample Preparation

Standard solution: each reference standard was accurately weighed and dissolved in methanol as the stock solutions. Afterward, appropriate amounts of eight stock solutions were mixed and diluted into a suitable working solution with methanol before it was used.

Sample solution: 100 g of PMRP was soaked for 1 h with 1000 mL 70% aqueous ethanol solution as soaking solvent and then refluxed for 2 h. After being filtered with gauze, the residue was refluxed twice with another 800 mL of 70% aqueous ethanol solution for 1h. Finally, the filtrates were mixed and evaporated to 0.5 g/mL as PMRPE in a rotary evaporator. The preparation method of PMRPW was the same as that of PMRPE, except that 70% aqueous ethanol solution was replaced with water. Appropriate PMRPE and PMRPW extract were diluted with 50% aqueous methanol solution to obtain 15 mg/mL solution, respectively (calculated as raw herbs). The solution was centrifuged at 13000 rpm for 10 min and filtered through a 0.22 µm membrane. An aliquot of 5 µL was injected for analysis.

### 3.3. LC System and Mass Spectrometry

LC analysis was performed on a Vanquish Flex UHPLC system (Thermo Fisher Scientific, Waltham, MA, USA) equipped with an HSS T3 column (2.1 mm × 100 mm, 1.8 μm, Waters Corporation, Milford, UK). The sample chamber and column temperatures were maintained at 10 °C and 35 °C, respectively. The gradient elution with mobile phase acetonitrile (A)—0.1% formic acid in water (B) was set as follows: 5–15% (A) from 0 to 4 min; 15–50% (A) from 4 to 10 min; 50–60% (A) from 10 to 15 min; 60–95% (A) from 15 to 25 min; 95% (A) from 25 to 28 min; 95–5% (A) from 28 to 28.1 min; 5% (A) from 28.1 to 31 min. The flow rate was 0.3 mL/min, and the injection volume was 5 μL.

A Q-Exactive Orbitrap mass spectrometry instrument (Thermo Fisher Scientific, USA) was used to identify the constituents of PMRPE and PMRPW in negative modes. The mass spectrometer was set with the following parameters: spray voltage, 3.0 kV; capillary temperature, 350 °C; auxiliary gas heater temperature, 350 °C; sheath gas flow rate, 35 Arb; auxiliary gas flow rate, 10 Arb; S-lens RF level, 55 V. The full scan and fragment spectra were collected at a resolution of 70,000 and 17,500, respectively. Full scan spectra were measured in a range from *m*/*z* 80 to 1200. The automatic gain control (AGC) target and maximum injection time (IT) were 3 × 10^6^ ions capacity and 50 ms, respectively. For the dd MS^2^ mode, the automatic gain control (AGC) target and maximum injection time (IT) were 1 × 10^5^ ions capacity and 50 ms. In each cycle, the top 10 precursor ions were chosen for fragmentation at collision energy (CE) of 20, 40 and 60 V. Data were analyzed by using Xcalibur™ version 2.2.1 and TraceFinder 4.1 version (Thermo Fisher Scientific, Waltham, MA, USA).

## 4. Conclusions

In this study, a rapid, sensitive, and specific analytical method was established using UHPLC-Q-Exactive Orbitrap-MS to identify the chemical constituents of PMRP. A total of 103 compounds, including 24 anthraquinones, 21 stilbenes, 15 phenolic acids, 14 flavones, and 29 other types were identified or tentatively characterized. There were 101 components in PMRPE and 91 components in PMRPW. Moreover, we have compared the peak areas of several significant components in PMRPW and PMRPE. The results showed that PMRPE has a higher content of anthraquinones and stilbenes than that of PMRPW. Previous studies have suggested that the hepatotoxicity of ethanol extract was stronger than that of water extract, indicating that anthraquinones and stilbenes might be the crucial xenobiotic components of liver injury induced by PMRP. Meanwhile, the specific toxicity compounds and mechanisms of hepatotoxicity also need further exploration. Considering the complex absorption and metabolism after oral administration, the characterization of PMRP’s composition in vitro research was not enough. Therefore, the identification of chemical constituents in vivo and the verification of hepatotoxicity mechanisms of PMRP are still under investigation. In conclusion, the profiles of the constituents provide more information to understand PMRP from a chemical viewpoint and establish a substantial basis for further studies. The results also demonstrated that the novel method would be meaningful to the characterization of components in other botanical extracts.

## Figures and Tables

**Table 1:** Chemical constituents identified in PMRP by UHPLC-Q-Exactive Orbitrap-MS.

NO.	RT (min)	Identification	Molecular Formula	Measured Mass [M−H]^−^	Accuracy Mass[M−H]^−^	Error (ppm)	Characteristic Fragment Ions	Source
Anthraquinones and derivatives								
30	5.88	Physcion-8-*O*-(6’-*O*- malonyl)-hexose	C_26_H_26_O_12_	529.13391	529.13405	−0.269	366.07205[M-H-C_6_H_11_O_5_]^−^ 348.06122[M-H-C_6_H_11_O_5_-H_2_O]^−^ 320.06482[M-H-C_6_H_11_O_5_-H_2_O-CO]^−^	PMRPW, PMRPE
33	6.09	Rumejaposide D ^a^	C_21_H_22_O_11_	449.10751	449.10784	−0.730	287.04864[M-H-C_6_H_10_O_5_]^−^ 269.04453[M-H-C_6_H_10_O_5_-H_2_O]^−^ 259.06021[M-H-C_6_H_10_O_5_-CO]^−^	PMRPW, PMRPE
57	8.41	Di-emodin-Di-glucoside ^a^	C_42_H_42_O_18_	833.22919	833.22874	0.539	671.17426[M-H-C_6_H_10_O_5_]^−^ 509.12094[M-H-2C_6_H_10_O_5_]^−^ 253.04974[M-H-2C_6_H_10_O_5_-C_15_H_12_O_4_]^−^	PMRPW
58	8.86	Isomer emodin-8-*O*-(6’-*O*-acetyl)-*β*-d- glucoside	C_23_H_22_O_11_	473.10638	473.10784	−3.081	311.05423[M-H-C_6_H_10_O_5_]^−^ 283.06085[M-H-C_6_H_10_O_5_-CO]^−^ 255.06544[M-H-C_6_H_10_O_5_-2CO]^−^	PMRPW, PMRPE
77	10.83	Citreorosein-*O*-glucoside ^a^	C_21_H_20_O_11_	447.09048	447.09219	−3.820	300.02811[M-H-C_6_H_10_O_4_]^−^ 268.03757[M-H-C_6_H_10_O_4_-2O]^−^ 240.04250[M-H-C_6_H_10_O_4_-CO]^−^	PMRPW, PMRPE
78	10.95	Chrysophanol ^b^	C_15_H_10_O_4_	253.05020	253.04954	2.627	225.05373[M-H-CO]^−^ 197.56078[M-H-2CO]^−^ 181.06459[M-H-CO_2_-CO]^−^	PMRPW, PMRPE
84	11.97	2-Acetylemodin-8-*O-β*-d-glucoside	C_23_H_22_O_11_	473.10583	473.10784	−4.424	311.05438[M-H-C_6_H_10_O_5_]^−^ 269.04575[M-H-C_6_H_10_O_5_-C_2_H_2_O]^−^ 241.04889[M-H-C_6_H_10_O_5_-C_2_H_2_O-CO]^−^	PMRPW, PMRPE
85	12.12	Emodin-8-*O-β*-d-glucoside ^b^	C_21_H_20_O_10_	431.09637	431.09727	−2.095	269.04520[M-H-C_6_H_10_O_5_]^−^ 241.04926[M-H-C_6_H_10_O_5_-CO]^−^ 225.05426[M-H-C_6_H_10_O_5_-CO_2_]^−^	PMRPW, PMRPE
86	12.35	Emodin-*O*-glucoside-gallate ^a^	C_28_H_24_O_14_	583.10736	583.10823	−0.872	269.04562[M-H-C_6_H_10_O_5_-C_7_H_4_O_4_]^−^ 225.05466[M-H-C_6_H_10_O_5_-C_7_H_4_O_4_-CO_2_]^−^	PMRPE
87	13.00	6-Carboxyl emodin ^a^	C_16_H_10_O_7_	313.03448	313.03428	0.642	269.04544[M-H-CO_2_]^−^ 241.05034[M-H-CO_2_-CO]^−^ 225.95458[M-H-2CO_2_]^−^	PMRPW, PMRPE
89	13.54	Physcion-8-*O-β*-d-glucoside	C_22_H_22_O_10_	445.11273	445.11292	−0.434	283.06104[M-H-C_6_H_10_O_5_]^−^ 255.06458[M-H-C_6_H_10_O_5_-CO]^−^ 239.06963[M-H-C_6_H_10_O_5_-CO_2_]^−^	PMRPW, PMRPE
90	13.65	Physcion ^b^	C_16_H_12_O_5_	283.06113	283.06010	3.639	268.03760[M-H-CH_3_]^−^ 240.04179[M-H-CH_3_-CO]^−^ 212.04668[M-H-CH_3_-2CO]^−^	PMRPW, PMRPE
91	14.09	Citreorosein	C_1_5H_10_O_6_	285.04041	285.03936	3.668	257.04422[M-H-CO]^−^ 241.04965[M-H-CO_2_]^−^ 227[M-H-CO-CH_2_O]^−^	PMRPW, PMRPE
92	14.59	Chrysophanol anthrone ^a^	C_15_H_12_O_3_	239.07027	239.06989	−1.593	210.89307[M-H-CO]^−^ 182.89648[M-H-2CO]^−^	PMRPE
93	14.69	Questinol	C_16_H_12_O_6_	299.05493	299.05501	−0.283	268.03696[M-H-CH_3_O]^−^ 253.04982[M-H-CH_3_O-CH_3_]^−^ 240.04204[M-H-CH_3_O-CO]^−^	PMRPW, PMRPE
94	14.87	Hydroxyl-rhein ^a^	C_15_H_8_O_7_	299.01867	299.01863	0.070	255.02777[M-H-CO_2_]^−^ 227.03313[M-H-CO_2_-CO]^−^ 199.03928[M-H-CO_2_-2CO]^−^	PMRPW, PMRPE
95	15.94	Digitolutein ^a^	C_16_H_12_O_4_	267.06601	267.06519	3.088	224.04675[M-H-CO-CH_3_]^−^ 220.53163[M-H-CO-H_2_O]^−^ 149.02373[M-H-2CO-CH_2_O-CH_2_-H_2_O]^−^	PMRPE
96	16.13	Isomer Citreorosein	C_15_H_10_O_6_	285.04041	285.03936	3.668	257.04462[M-H-CO]^−^ 241.04955[M-H-CO_2_]^−^ 211.03883[M-H-CO_2_-CH_2_O]^−^	PMRPW, PMRPE
98	17.02	Emodin anthrone ^a^	C_15_H_12_O_4_	255.06572	255.06519	2.096	240.04176[M-H-CH_3_]^−^ 225.05334[M-H-CH_2_O]^−^ 212.04684[M-H-CH_3_-CO]^−^	PMRPW, PMRPE
99	17.05	Isomer physcion	C_16_H_12_O_5_	283.06067	283.06010	2.014	240.04173[M-H-CH_3_-CO]^−^ 268.03635[M-H-CH_3_]^−^	PMRPW, PMRPE
100	18.20	Emodin-3- ethyl ether	C_17_H_14_O_5_	297.07452	297.07575	0.606	282.05472[M-H-CH_3_]^−^ 269.08282[M-H-CO]^−^ 254.05786[M-H-C_2_H_5_-CH_2_]^−^	PMRPW, PMRPE
101	18.86	2-Acetylemodin	C_17_H_12_O_6_	311.05408	311.05501	−3.004	296.03165[M-H-CH_3_]^−^ 283.06100[M-H-CO]^−^ 269.04504[M-H-C_2_H_2_O]^−^	PMRPW, PMRPE
102	19.52	Isomer 2-acetylemodin	C_17_H_12_O_6_	311.05426	311.05501	−2.426	283.06119[M-H-CO]^−^ 269.06583[M-H-C_2_H_2_O]^−^ 240.04160[M-H-2CO-CH_3_]^−^	PMRPW, PMRPE
103	20.37	Emodin ^b^	C_15_H_10_O_5_	269.04514	269.04445	2.565	241.04941[M-H-CO]^−^ 213.05467[M-H-2CO]^−^ 225.05450[M-H-CO_2_]^−^	PMRPW, PMRPE
Stilbenes and derivatives								
29	5.24	Polygonumosides C	C_40_H_44_O_19_	827.23828	827.23931	−1.239	421.11276[M-H-C_20_H_22_O_9_]^−^ 259.06009[M-H-C_20_H_22_O_9_-C_6_H_10_O_5_]^−^ 241.04927[M-H-C_20_H_22_O_9_- C_6_H_10_O_5_-H_2_O]^−^	PMRPW
37	6.69	Isomer 3,4,5,4’-tetrahydroxystilbene	C_14_H_12_O_4_	243.06465	243.06519	−2.202	225.05467[M-H-H_2_O]^−^ 215.06952[M-H-CO]^−^ 197.05991[M-H-H_2_O-CO]^−^	PMRPW, PMRPE
41	7.07	Rhapontin ^a^	C_21_H_24_O_9_	419.13242	419.13366	−2.955	257.07297[M-H-C_6_H_10_O_5_]^−^ 239.03387[M-H-C_6_H_10_O_5_-H_2_O]^−^ 227.03279[M-H-C_6_H_10_O_5_-CH_2_O]^−^	PMRPW, PMRPE
44	7.31	Tetrahydroxystilbene-*O*-di-glucoside	C_26_H_32_O_14_	567.17004	567.17083	−1.396	243.06516[M-H-2C_6_H_10_O_5_]^−^ 225.05428[M-H-2C_6_H_10_O_5_-H_2_O]^−^ 197.06007[M-H-2C_6_H_10_O_5_-H_2_O-CO]^−^	PMRPW, PMRPE
45	7.38	*β-d*-glucoside,4-[2,3-dihydro-3-(hydroxymethyl)-5-(3-hydroxypropyl)-7-methoxy-2-yl]-2-methoxyphenyl ^a^	C_26_H_34_O_11_	521.20062	521.20174	−2.145	359.147229[M-H-C_6_H_10_O_5_]^−^ 313.10608[M-H-C_6_H_10_O_5_-H_2_O-CO]^−^	PMRPW, PMRPE
47	7.76	Resveratrol ^a^	C_14_H_12_O_3_	227.07057	227.07027	1.318	185.05931[M-H-H_2_O-CH_2_]^−^ 170.97986[M-H-H_2_O-2CH_2_]^−^ 143.04947[M-H-C_4_H_4_O_2_]^−^	PMRPW, PMRPE
48	7.67	Isomer Tetrahydroxystilbene-*O*-di-glucoside	C_26_H_32_O_14_	567.17059	567.17083	−0.427	243.06509[M-H-2C_6_H_10_O_5_]^−^ 225.05261[M-H-2C_6_H_10_O_5_-H_2_O]^−^	PMRPW, PMRPE
50	7.89	3,4,5,4’-Tetrahydroxystilbene	C_14_H_12_O_4_	243.06517	243.06519	−0.063	225.05469[M-H-H_2_O]^−^ 197.05965[M-H-H_2_O-CO]^−^ 169.06514[M-H-H_2_O-2CO]^−^	PMRPW, PMRPE
52	7.94	2, 3, 5, 4′-Tetrahydroxystilbene-2-*O-β*-d-glucoside ^b^	C_20_H_22_O_9_	405.11670	405.11801	−0.885	243.06503[M-H-C_6_H_10_O_5_]^−^ 225.05450[M-H-C_6_H_10_O_5_-H_2_O]^−^ 215.07039[M-H-C_6_H_10_O_5_-CO]^−^	PMRPW, PMRPE
56	8.32	Multiflorumisides A ^a^	C_40_H_44_O_18_	811.24438	811.24439	−0.013	649.19263[M-H-C_6_H_10_O_5_]^−^ 405.11447[M-H-C_20_H_22_O_9_]^−^ 243.06512[M-H-C_20_H_22_O_9_-C_6_H_10_O_5_]^−^	PMRPW, PMRPE
61	9.17	Polygonumoside A	C_27_H_24_O_13_	555.11487	555.11332	2.797	393.05923[M-H-C_6_H_10_O_5_]^−^ 349.07019[M-H-C_6_H_10_O_5_-CO_2_]^−^ 300.99774[M-H-C_6_H_10_O_5_-C_6_H_4_O]^−^	PMRPW, PMRPE
64	9.53	Isomer polygonumoside A	C_27_H_24_O_13_	555.11432	555.11332	1.807	393.05942[M-H-C_6_H_10_O_5_]^−^ 349.06873[M-H-C_6_H_10_O_5_-CO_2_]^−^ 300.99670[M-H-C_6_H_10_O_5_-C_6_H_4_O]^−^	PMRPW, PMRPE
66	9.56	2,3,5,4′-Tetrahydroxystilbene-*O*-(malonyl)- *β*-d-glucoside	C_23_H_24_O_12_	491.11929	491.11840	1.807	329.09622[M-H-C_6_H_10_O_5_]^−^ 313.03226[M-H-C_6_H_10_O_5_-H_2_O]^−^ 285.04071[M-H-C_6_H_10_O_5_-H_2_O-CO]^−^	PMRPW, PMRPE
67	9.57	Tetrahydroxystilbene-*O*-(galloyl)-glucoside	C_27_H_26_O_13_	557.12933	557.12897	0.651	405.05499[M-H-C_7_H_4_O_4_]^−^ 243.06503[M-H-C_7_H_4_O_4_-C_6_H_10_O_5_]^−^ 225.05434[M-H-C_7_H_4_O_4_-C_6_H_10_O_5_-H_2_O]^−^	PMRPW, PMRPE
68	9.87	2,3,5,4’-Tetrahydroxystilbene-2-*O*-(2”-*O*-acetyl)-*β*-d-glucoside	C_22_H_24_O_10_	447.12930	447.12857	1.625	243.06511[M-H-C_6_H_10_O_5_-C_2_H_2_O]^−^ 225.05455[M-H-C_6_H_10_O_5_-C_2_H_2_O-H_2_O]^−^ 284.08289[M-H-C_6_H_11_O_5_]^−^	PMRPE
69	9.95	Piceatannol-3-*O-β*-d-(6″-*O*-galloyl)- glucoside	C_27_H_26_O_13_	557.12817	557.12897	−1.431	405.11728[M-H-C_7_H_4_O_4_]^−^ 243.06511[M-H-C_7_H_4_O_4_-C_6_H_10_O_5_]^−^ 225.05495[M-H-C_7_H_4_O_4_-C_6_H_10_O_5_-H_2_O]^−^	PMRPW, PMRPE
73	10.70	Tetrahydroxystilbene-*O*-(caffeoyl)-glucoside ^a^	C_29_H_28_O_12_	567.14502	567.14970	−8.256	243.06516[M-H-C_6_H_10_O_5_-C_9_H_6_O_3_]^−^	PMRPE
75	10.81	Polydatin ^a b^	C_20_H_22_O_8_	389.12457	389.12309	0.984	227.07018[M-H-C_6_H_10_O_5_]^−^ 209.05940[M-H-C_6_H_10_O_5_-H_2_O]^−^ 199.07462[M-H-C_6_H_10_O_5_-CO]^−^	PMRPW, PMRPE
76	10.83	Isorhapontigenin ^a^	C_15_H_14_O_4_	257.08127	257.08084	1.691	242.05462[M-H-CH_3_]^−^ 187.56930[M-H-CH_2_-2CO]^−^ 136.18150[M-H-C_7_H_5_O_2_]^−^	PMRPW, PMRPE
81	11.39	2,3,5,4′-Tetrahydroxystilbene-2-*O-β*-d-(2″-*O*-coumaroyl)-glucoside	C_29_H_28_O_11_	551.15112	551.15479	−3.668	389.10031[M-H-C_6_H_10_O_5_]^−^ 225.05389[M-H-C_6_H_10_O_5_-C_9_H_6_O_2_-H_2_O]^−^	PMRPE
88	13.17	Tetrahydroxystilbene-2-(feruloyl)- glucoside	C_30_H_30_O_12_	581.16583	581.16535	0.821	419.11136[M-H-C_6_H_10_O_5_]^−^ 405.21970[M-H-C_10_H_8_O_3_]^−^ 295.05981[M-H-C_6_H_10_O_5_-C_6_H_4_O_3_]^−^	PMRPW, PMRPE
Flavonoids and derivatives								
18	3.63	Liquiritigenin-glucoside-xyl/ara	C_26_H_30_O_13_	549.1604	549.16027	0.023	387.10541[M-H-C_6_H_10_O_5_]^−^ 369.09552[M-H-C_6_H_10_O_5_-H_2_O]^−^ 279.06604[M-H-C_6_H_10_O_5_-C_5_H_8_O_4_]^−^	PMRPW, PMRPE
28	4.79	Catechin	C_15_H_14_O_6_	289.07111	289.07066	1.541	151.03955[M-H-C_7_H_5_O_3_]^−^ 137.02376[M-H-C_8_H_7_O_3_]^−^ 123.04458[M-H-C_7_H_5_O_3_-CO]^−^ 109.02898[M-H-C_8_H_7_O_3_-C_2_H_2_O]^−^	PMRPW, PMRPE
31	5.93	Epicatechin	C_15_H_14_O_6_	289.07080	289.07066	0.468	151.03955[M-H-C_7_H_5_O_3_]^−^ 137.02376[M-H-C_8_H_7_O_3_]^−^ 123.04458[M-H-C_7_H_5_O_3_-CO]^−^ 109.02898[M-H-C_8_H_7_O_3_-C_2_H_2_O]^−^	PMRPW, PMRPE
35	6.36	Acetyl-epicatechin-*O*-glucoside ^a^	C_23_H_26_O_12_	493.13406	493.13405	0.015	330.07205[M-H-C_6_H_11_O_5_]^−^ 255.06543[M-H-C_6_H_10_O_5_-C_2_H_2_O_2_-H_2_O]^−^ 227.07016[M-H-C_6_H_10_O_5_-C_2_H_2_O_2_-H_2_O-CO]^−^	PMRPW, PMRPE
39	7.04	Hesperetin-7-*O*-glucoside ^a^	C_22_H_24_O_11_	463.12482	463.12349	2.876	419.13446[M-H-CO_2_]^−^ 256.07315[M-H-CO_2_-C_6_H_11_O_5_]^−^	PMRPW, PMRPE
49	7.73	Trihydroxy-dimethoxychalcone-*O*-glucoside	C_23_H_26_O_11_	477.13867	477.13914	−0.981	315.08606[M-H-C_6_H_10_O_5_]^−^ 297.07486[M-H-C_6_H_10_O_5_-H_2_O]^−^ 243.06522[M-H-C_6_H_10_O_5_-2CO-O]^−^	PMRPW, PMRPE
51	7.90	Epicatechin-*O*-gallate	C_22_H_18_O_10_	441.08109	441.08162	−3.706	289.07086[M-H-C_7_H_5_O_4_]^−^ 243.06519[M-H-C_7_H_5_O_4_-H_2_O-CO]^−^225.05489[M-H-C_7_H_5_O_4_-CO_2_]^−^ 169.01367[M-H-C_15_H_13_O_5_]^−^	PMRPE
55	8.19	Cirsimarin ^a^	C_23_H_24_O_11_	475.12415	475.12349	1.394	313.06976[M-H-C_6_H_10_O_5_]^−^ 285.07596[M-H-C_6_H_10_O_5_-CO]^−^ 242.05670[M-H-C_6_H_10_O_5_-CO_2_-2CH_2_]^−^	PMRPW, PMRPE
59	9.03	Epimedium	C_20_H_20_O_7_	371.11102	371.11253	−4.067	281.08215[M-H-C_4_H_10_O_2_]^−^ 161.02383[M-H-C_4_H_8_O_2_-C_7_H_4_O_2_]^−^	PMRPE
60	9.11	Kaempferol-3-*β*-d-glucoside	C_21_H_20_O_11_	447.09103	447.09219	−3.529	285.03925[M-H-C_6_H_10_O_5_]^−^ 257.04456[M-H-C_6_H_10_O_5_-CO]^−^ 229.04724[M-H-C_6_H_10_O_5_-CO]^−^	PMRPW, PMRPE
63	9.49	Quercetin	C15H10O7	301.03424	301.03428	−0.130	283.03264[M-H-H2O]^−^ 273.04050[M-H-CO]^−^ 255.02896[M-H-CO-H2O]^−^	PMRPW, PMRPE
65	9.55	Kaempferol ^a^	C_15_H_10_O_6_	285.04022	285.03936	3.001	241.04958[M-H-CO_2_]^−^ 257.67783[M-H-CO]^−^	PMRPW, PMRPE
70	10.27	Kaempferol-*O*-glucoside-rhamnose ^a^	C_27_H_30_O_15_	593.14893	593.15010	−1.967	269.04529[M-H-2C_6_H_10_O_5_]^−^ 225.05469[M-H-2C_6_H_10_O_5_-CO_2_]^−^ 241.04984[M-H-2C_6_H_10_O_5_-CO]^−^	PMRPW, PMRPE
74	10.76	Dihydroquercetin	C_15_H_12_O_7_	303.04868	303.04929	−2.109	151.03946[M-H-CO_2_-C_6_H_4_O_2_]^−^ 153.01883[M-H-C_8_H_7_O_3_]^−^ 125.02396[M-H-C_8_H_7_O_3_-CO]^−^	PMRPW, PMRPE
Phenolic acids and derivatives								
9	1.99	Gallic acid ^b^	C_7_H_6_O_5_	169.01358	169.01315	2.546	125.02383[M-H-CO_2_]^−^ 107.01351[M-H-CO_2_-H_2_O]^−^ 97.02921[M-H-CO_2_-CO]^−^	PMRPW, PMRPE
10	2.06	Gallic acid-*O*- glucoside	C_13_H_16_O_10_	331.06595	331.06597	−0.070	169.01357[M-H-C_6_H_10_O_5_]^−^ 125.02380[M-H-C_6_H_10_O_5_-CO_2_]^−^	PMRPW, PMRPE
12	2.75	Dihydroxy-benzoic acid ^a^	C_7_H_6_O_4_	153.01859	153.01824	2.319	125.02429[M-H-CO]^−^ 109.02917[M-H-CO_2_]^−^	PMRPW, PMRPE
14	2.83	Galloyl-glycerol ^a^	C_10_H_12_O_7_	243.04993	243.04993	0.004	169.01321[M-H-C_3_H_6_O_2_]^−^ 125.02386[M-H-C_3_H_6_O_2_-CO_2_]^−^ 118.96574[M-H-C_3_H_6_O_2_-3O]^−^	PMRPW, PMRPE
15	2.90	Vanillic acid ^a^	C_8_H_8_O_4_	167.03433	167.03389	−0.331	137.02341[M-H-CH_2_O]^−^ 123.04459[M-H-CO_2_]^−^	PMRPW, PMRPE
16	3.30	Isomer Dihydroxy- benzoic acid	C_7_H_6_O_4_	153.01859	153.01824	−0.230	137.45282[M-H-O]^−^ 125.02434[M-H-CO]^−^ 109.02898[M-H-CO_2_]^−^	PMRPW, PMRPE
17	3.34	Protocatechuic acid-*O*-glucoside	C_13_H_16_O_9_	315.06995	315.07106	−3.518	153.01865[M-H-C_6_H_10_O_5_]^−^ 109.02901[M-H-C_6_H_10_O_5_-CO_2_]^−^	PMRPW, PMRPE
19	3.71	Caffeic acid ^a^	C_9_H_8_O_4_	179.03412	179.03389	2.093	135.04448[M-H-CO_2_]^−^ 107.04977[M-H-C_3_H_4_O_2_]^−^	PMRPW, PMRPE
20	3.75	1-(5-Methylfuran-2-yl) ethanone ^a^	C_7_H_8_O_2_	123.04462	123.04406	4.584	108.02116[M-H-CH_3_]^−^ 95.01338[M-H-CO]^−^ 79.05503[M-H-CO_2_]^−^	PMRPW, PMRPE
21	4.16	Veratric acid ^a^	C_9_H_10_O_4_	181.05014	181.04954	2.567	137.02396[M-H-CO_2_]^−^ 122.03658[M-H-2CH_2_O]^−^ 107.04949[M-H-CO-CH_2_O]^−^	PMRPW, PMRPE
25	4.56	3-Hydroxybenzoic acid	C_7_H_6_O_3_	137.02371	137.02332	2.842	119.01297[M-H-H_2_O]^−^ 93.03405[M-H-CO_2_]^−^	PMRPW, PMRPE
26	4.75	Coumaric acid ^a^	C_9_H_8_O_3_	163.03937	163.03897	2.4500	119.04978[M-H-CO_2_]^−^ 134.91408[M-H-CO]^−^ 107.04973[M-H-C_2_O_2_]^−^	PMRPW, PMRPE
27	4.77	2-Methyl gallic acid ^a^	C_8_H_8_O_5_	183.02893	183.02880	0.711	168.00560[M-H-CH_3_]^−^ 139.00296[M-H-CO_2_]^−^ 111.00824[M-H-CO_2_-CO]^−^	PMRPW, PMRPE
34	6.24	Methyl gallate ^a^	C_8_H_8_O_4_	167.03424	167.03389	2.124	151.00310[M-H-CH_3_]^−^ 125.02368[M-H-C_2_H_2_O]^−^107.01314[M-H-C_2_H_2_O_2_]^−^	PMRPW, PMRPE
40	7.06	Syringic acid ^a^	C_9_H_10_O_5_	197.04417	197.04445	−1.420	169.01370[M-H-CO]^−^ 125.02389[M-H-CO-CO_2_]^−^	PMRPE
Others								
1	0.73	L-Arginine	C_6_H_14_N_4_O_2_	173.10316	173.10330	−0.821	131.08197[M-H-CN_2_H_2_]^−^ 114.05562[M-H-NH-CO_2_]^−^	PMRPW, PMRPE
2	0.79	Glucose	C_6_H_12_O_6_	179.05534	179.05501	1.818	161.06087[M-H-H_2_O]^−^ 131.03432[M-H-H_2_O-CH_2_O]^−^ 85.02903[M-H-CH_2_O-4O]^−^	PMRPW, PMRPE
3	0.82	L-Threonine	C_4_H_9_NO_3_	118.05041	118.04987	4.577	74.02446[M-H-CO_2_]^−^ 59.01369[M-H-CO_2_-CH_3_]^−^	PMRPW, PMRPE
4	0.85	(2S)-2-Hydroxybutanedioic acid	C_4_H_6_O_5_	133.01361	133.01315	3.460	115.00312[M-H-H_2_O]^−^ 89.02412[M-H-CO_2_]^−^ 71.01358[M-H-H_2_O-CO_2_]^−^	PMRPW, PMRPE
5	1.29	Citric acid ^a^	C_6_H_8_O_7_	191.01889	191.01863	1.366	129.01921[M-H-CO_2_-H_2_O]^−^ 111.00829[M-H-CO_2_-2H_2_O]^−^ 87.00838[M-H-2CO_2_-O]^−^	PMRPW, PMRPE
6	1.39	L-Tyrosine ^a^	C_9_H_11_NO_3_	180.06575	180.06552	1.279	163.03926[M-H-OH]^−^ 137.02368[M-H-NH-CO]^−^ 119.04951[M-H-CO_2_-OH]^−^	PMRPW, PMRPE
7	1.40	3-*O*-feruloylquinic acid ^a^	C_17_H_20_O_9_	367.10272	367.10236	0.985	277.07294[M-H-COOH-CH_2_O-H_2_O]^−^ 157.03020[M-H-C_10_H_8_O_3_-2OH]^−^	PMRPW, PMRPE
8	1.43	Leucine	C_6_H_13_NO_2_	130.08676	130.08626	3.881	85.02912[M-H-COOH]^−^ 88.04015[M-H-3CH_2_]^−^	PMRPW, PMRPE
11	2.50	3,5-Dihydroxy-2- methyl-4hydro-pyran-4-one	C_6_H_6_O_4_	141.01833	141.01824	0.673	112.95596[M-H-CO]^−^ 97.02898[M-H-CO_2_]^−^ 69.03445[M-H-CO_2_-H_2_O]^−^	PMRPW, PMRPE
13	2.79	5-Hydroxymethylfurfural	C_6_H_6_O_3_	125.02386	125.02332	2.795	97.02918[M-H-CO]^−^ 81.03435[M-H-CO_2_]^−^	PMRPW, PMRPE
22	4.35	Altechromone A ^a^	C_11_H_10_O_3_	189.05476	189.05462	0.737	174.03186[M-H-CH_3_]^−^ 161.06018[M-H-CO]^−^ 146.03635[M-H-CO-CH_3_]^−^	PMRPW, PMRPE
23	4.52	Acetyl 1-methyl-1- acetoxyethyl peroxide ^a^	C_7_H_12_O_5_	175.06026	175.06010	0.914	160.97757[M-H-CH_2_]^−^ 146.96054[M-H-2CH_2_]^−^ 115.03953[M-H-2CH_2_O]^−^	PMRPW, PMRPE
24	4.56	2-Vinyl-1H-indole-3-carboxylic acid	C_11_H_9_NO_2_	186.05539	186.05496	2.338	142.06551[M-H-CO_2_]^−^ 159.93617[M-H-C_2_H_3_]^−^ 116.05013[M-H-C_2_H_2_-CO_2_]^−^	PMRPE
32	6.01	P-hydroxybenzal-dehyde ^a^	C_7_H_6_O_2_	121.0289	121.02841	4.082	93.03405[M-H-CO]^−^	PMRPW, PMRPE
36	6.54	Vanillin ^a^	C_8_H_8_O_3_	151.03902	151.03897	0.327	136.01607[M-H-CH_3_]^−^ 123.04456[M-H-CO]^−^ 107.04993[M-H-CO_2_]^−^	PMRPW, PMRPE
38	7.03	6-Methoxyl-2-Acetyl-3-methyljuglone-8-*O-β*-d-glucoside	C20H22O10	421.11276	421.11292	−0.388	259.06027[M-H-C6H10O5]^−^ 241.04961[M-H-C6H10O5-H2O]^−^ 213.05441[M-H-C6H10O5-H2O-CO]^−^	PMRPW, PMRPE
42	7.13	Nudiposide ^a^	C_27_H_36_O_12_	551.21313	551.21230	1.501	389.15720[M-H-C_6_H_10_O_5_]^−^ 359.11261[M-H-C_6_H_10_O_5_-2CH_2_O]^−^ 341.09985[M-H-C_6_H_10_O_5_-2CH_2_O-H_2_O]^−^	PMRPW, PMRPE
43	7.21	(+)-lyoniresinol-2α-*O-β*-glucoside ^a^	C_28_H_38_O_13_	581.22284	581.22287	−0.994	419.16949[M-H-C_6_H_10_O_5_]^−^389.12219[M-H-C_6_H_10_O_5_-CH_2_O]^−^359.11096[M-H-C_6_H_10_O_5_-2CH_2_O]^−^	PMRPW, PMRPE
46	7.40	Isomer Altechromone A	C_11_H_10_O_3_	189.0547	189.05462	0.420	174.03166[M-H-CH_3_]^−^ 161.06047[M-H-CO]^−^ 147.04448[M-H-CO-CH_2_]^−^	PMRPW, PMRPE
53	8.08	Cinnamyl-galloyl-*O*- glucoside ^a^	C_22_H_22_O_11_	461.10611	461.10784	−3.747	417.11594[M-H-CO_2_]^−^ 254.05766[M-H-CO_2_-C_6_H_11_O_5_]^−^	PMRPW, PMRPE
54	8.11	2-Methyl-5-carboxymethyl-7-hydroxychromone ^a^	C_12_H_10_O_5_	233.04443	233.04555	−0.085	205.04994[M-H-CO]^−^ 191.03783[M-H-CO-CH_2_]^−^ 161.02485[M-H-CO-CO_2_]^−^	PMRPE
62	9.24	Trans-N-caffeoyltyramine ^a^	C_17_H_17_NO_4_	298.10773	298.10738	1.159	135.04459[M-H-C_9_H_7_O_3_]^−^ 178.04970[M-H-C_8_H_8_O]^−^ 148.05200[M-H-C_8_H_6_O-OH]^−^	PMRPW, PMRPE
71	10.30	Noreugenin ^a^	C_10_H_8_O_4_	191.03399	191.03389	0.549	149.02406[M-H-CO-CH_2_]^−^ 147.04459[M-H-CO_2_]^−^	PMRPW, PMRPE
72	10.61	1,2-Dihydroxypropane-1-(4-hydroxy-phenyl) ^a^	C_9_H_12_O_3_	167.07063	167.07053	1.552	152.04729[M-H-CH_3_]^−^ 138.92862[M-H-CO]^−^	PMRPW, PMRPE
79	10.98	N-trans-Feruloyl tyramine	C_18_H_19_NO_4_	312.12286	312.12303	−0.559	190.05000[M-H-C_7_H_6_O_2_]^−^ 178.05019[M-H-C_8_H_6_O_2_]^−^ 148.05235[M-H-C_9_H_10_NO_2_]^−^	PMRPW, PMRPE
80	11.34	Trans-N-Feruloyl-3-*O*-methyldopamine	C_19_H_21_NO_5_	342.13211	342.13360	−4.353	327.10962[M-H-CH_3_]^−^ 178.05003[M-H-C_9_H_8_O_3_]^−^	PMRPW, PMRPE
82	11.43	Thunberginol C-6- *O-β*-d-glucoside ^a^	C21H22O10	433.11395	433.11292	2.371	271.06082[M-H-C6H10O5]^−^ 253.05016[M-H-C6H10O5-H2O]^−^ 243.06531[M-H-C6H10O5-CO]^−^	PMRPW, PMRPE
83	11.87	Torachrysone ^a^	C_14_H_14_O_4_	245.08078	245.08084	−0.226	230.05690[M-H-CH_3_]^−^ 215.03368[M-H-CH_2_O]^−^ 159.04398[M-H-CH_2_O-CH_2_-C_2_H_2_O]^−^	PMRPE
97	16.62	3,8-Dihydroxy-1- methoxyxanthone ^a^	C14H10O5	257.04535	257.04445	3.502	239.03391[M-H-H2O]^−^ 229.04854[M-H-CO]^−^ 211.03917[M-H-CO-H2O]^−^	PMRPW, PMRPE

Note: PMRPE: Ethanol extract of Polygoni Multiflori Radix Praeparata; PMRPW: Water extract of Polygoni Multiflori Radix Praeparata; ^a^ means first reported in PMRP; ^b^ means components compared with standards.

**Table 2:** The peak area of potentially xenobiotic compounds in PMRPW and PMRPE.

Peak No.	RT	Compound Name	Molecular Formula	Area		Percentage of the Area		Mechanisms of Hepatotoxicity
				PMRPW	PMRPE	PMRPW	PMRPE	
9	1.99	Gallic acid	C_7_H_6_O_5_	4,237,639,440 *	3,642,690,762	99.93%	99.91%	CYP1A2 ↓; Caspase-3 ↑
28	4.79	Catechin	C_15_H_14_O_6_	22,672,980 *	16,781,588	95.54%	96.05%	UGT1A6 ↓; UGT2B1 ↓
31	5.93	Epicatechin	C_15_H_14_O_6_	10,315,546 *	6,669,699	92.05%	90.44%	Caspase-3 ↑; UGT1A6 ↑; UGT2B1 ↓
47	7.76	Resveratrol	C_14_H_12_O_3_	5,525,111	6,868,739 *	76.05%	74.84%	Cyp7a1 ↑
52	7.94	THSG	C_20_H_22_O_9_	32,436,422	49,753,744 *	96.08%	95.11%	Cyp7a1 ↑; Hmgcr ↑ CytP-450 ↓; UDP ↓
85	12.12	EG	C_21_H_20_O_10_	5,266,457	10,448,815 *	97.11%	96.42%	UDP ↓; CYP2A ↓; UGT1A1 ↓; CYP3A4 ↓
90	13.65	Physcion	C_16_H_12_O_5_	187,344,509	394,975,616 *	94.04%	95.77%	Cyp8b1 ↓; Cyp7a1 ↑
103	20.37	Emodin	C_15_H_10_O_5_	4,512,341,510	773,950,089 *	99.71%	99.28%	CytP-50 ↓; Cyp8b1 ↓ Cyp7a1 ↑; CYP3A4 ↓

*: means the peak area of this group is higher than the other group; “↑”: means promoting expression; “↓”: means inhibiting expression.

## Supplementary Materials

The following are available online, Figure S1: Chemical structures of compounds identified in PMRP.

## Sample Availability

Samples of the compounds are available from the authors.
