# Supplementary material for: High-Throughput Identification of Organic Compounds from Polygoni Multiflori Radix Praeparata (Zhiheshouwu) by UHPLC-Q-Exactive Orbitrap-MS
Source: Molecules. 2021 Jun 29;26(13):3977. doi: 10.3390/molecules26133977 (PMC8428211; doi:10.3390/molecules26133977)
Supplement: Supplementary file 1 [file molecules-26-03977-s001.zip › molecules-1243606-supplementary.pdf]

High-Throughput Identification of Organic Compounds from Polygoni Multiflori Radix Praeparata (*Zhiheshouwu*) by UHPLC-Q-Exactive Orbitrap-MS

Shao-Yun Wang, Xiao-Zhu Sun, Shuo An, Fang Sang, Yun-Li Zhao * and Zhi-Guo Yu *

School of Pharmacy, Shenyang Pharmaceutical University, Shenyang 110016, Liaoning Province, P. R. China; wsy17862968828@163.com (S.-Y.W.); sxz13591173561@163.com (X.-Z.S.); a13831181394@163.com (S.A.); sangfang03@163.com (F.S.)

* Correspondence: yunli76@163.com (Y.-L.Z.); zhiguo-yu@163.com (Z.-G.Y.)

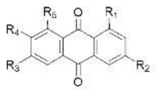

Compound	R1	R2	R3	R4	R5
Emodin-8-O-(6'-O-acetyl)-β-D-glucoside	OH	CH3	OH	H	Glc-Ace
Chrysophanol	OH	CH3	H	H	OH
Emodin-O-glucoside-gallate	Gallate	CH3	OH	H	Glc
6-Carboxyl emodin	OH	CH2COOH	OH	H	OH
Physcion-8-O-β-D-glucoside	Glc	OCH3	CH3	H	OH
Physcion	OH	OCH3	CH3	H	OH
Citreorosein	OH	CH2OH	OH	H	OH
Questinol	OH	CH2OH	OH	H	OCH3
Hydroxyl-rhein	OH	COOH	OH	H	OH
Digitolutein	OCH3	OH	CH2OH	H	OH
Emodin-3-ethyl ether	OH	OCH2CH3	CH3	H	OH
2-acetylemodin	OH	OH	CH3	COCH3	OH
Emodin	OH	CH3	OH	H	OH
Emodin-8-O-β-D-glucoside	OH	CH3	OH	H	Glc
Physcion-8-O-(6'-O-malonyl)-hexose	Glc-Mal	OCH3	CH3	H	OH
Citreorosein -O-glucoside	OH	CH2OH	OH	H	Glc

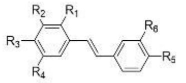

Compound	R1	R2	R3	R4	R5	R6
3,4,5,4'-tetrahydroxystilbene	H	OH	OH	OH	OH	H
Rhapontin	H	OH	H	Glc	OCH3	OH
Tetrahydroxystilbene-O-di-glucoside	Glc	OH	H	Glc	OH	H
Resveratrol	H	OH	H	OH	OH	H
(Z)-2, 3, 5, 4′-Tetrahydroxystilbene-2-O-β-D-glucoside	Glc	OH	H	OH	OH	H
2,3,5,4'-Tetrahydroxystilbene-O-(malonyl)-β-D-glucoside	Mal	OH	H	Glc	OH	H
Tetrahydroxystilbene-O-(galloyl)-glucoside	Glc	OH	H	Gal	OH	H
Piceatannol-3-O-β-D-(6″-O-galloyl)-glucoside	H	OH	H	OH	OH	Glc-Gal
Tetrahydroxystilbene-O-(caffeoyl)-glucoside	Glc	OH	H	Caf	OH	H
Polydatin	H	O-Glc	H	OH	OH	H
Isorhapontigenin	H	OH	H	OH	OH	H
2,3,5,4'-tetrahydroxystilbene-2-O-β-D-(2″-O-coumaroyl)-glucoside	Glc-Cou	OH	H	OH	OH	H
2,3,5 ,4'-tetrahydroxystilbene-2-O-(2"-O-acetyl)-β-D-glucoside	Glc-Ace	OH	H	OH	OH	H
Tetrahydroxystilbene-2-(feruloyl)-glucoside	Glc	OH	H	Fer	OH	H

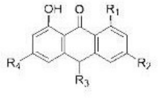

Compound	R1	R2	R3	R4
Rumejaposide D	OH	OH	Glc	CH2OH
Di-Emodin anthrone-Di-glucoside	Glc	H	H	EA-Glc
Chrysophanol anthrone	H	H	H	CH3
Emodin anthrone	OH	OH	H	CH3

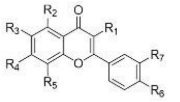

Compound	R1	R2	R3	R4	R5	R6	R7
Cirsimarin	H	OH	OCH3	OCH3	H	Glc	H
Liquiritigenin-glucoside-xyl/ara	H	H	H	Xyl	H	Glc	H
Hesperetin-7-O-glucoside	H	OH	H	Glc	H	OCH3	OH
Epimedium	H	OH	H	OH	C5H11O	OH	H
Kaempferol-3-β-D-glucoside	Glc	OH	H	OH	H	OH	H
Quercetin	OH	OH	H	OH	H	OH	OH
Kaempferol	OH	OH	H	OH	H	OH	H
Kaempferol-O-hexose-rhamnose	Glc	OH	H	Rha	H	OH	H
Dihydroquercetin	OH	OH	H	OH	H	OH	OH

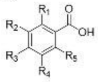

Compound	R1	R2	R3	R4	R5
Gallic acid	H	OH	OH	OH	H
Gallic acid-O-glucoside	H	OH	OH	Glc	H
Dihydroxy-benzoic acid	OH	H	H	H	OH
Vanillic acid	H	OCH3	OH	H	H
Protocatechuic acid-O-glucoside	H	Glc	OH	H	H
Veratric acid	H	OCH3	OCH3	H	H
3-Hydroxybenzoic acid	H	OH	H	H	H
2Mmethyl gallic acid	CH3	OH	OH	OH	H
Syringic acid	H	OCH3	OH	OCH3	H

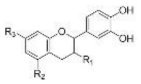

Compound	R1	R2	R3
Catechin	OH	OH	OH
Acetyl-epicatechin-O-glucoside	OH	Glc	Ace
Epicatechin-O-gallate	Gal	OH	OH

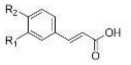

Compound	R1	R2
Caffeic acid	OH	OH
Coumaric acid	H	OH

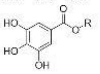

Compound	R
Galloyl-glycerol	Gly
Syringic acid	OCH3

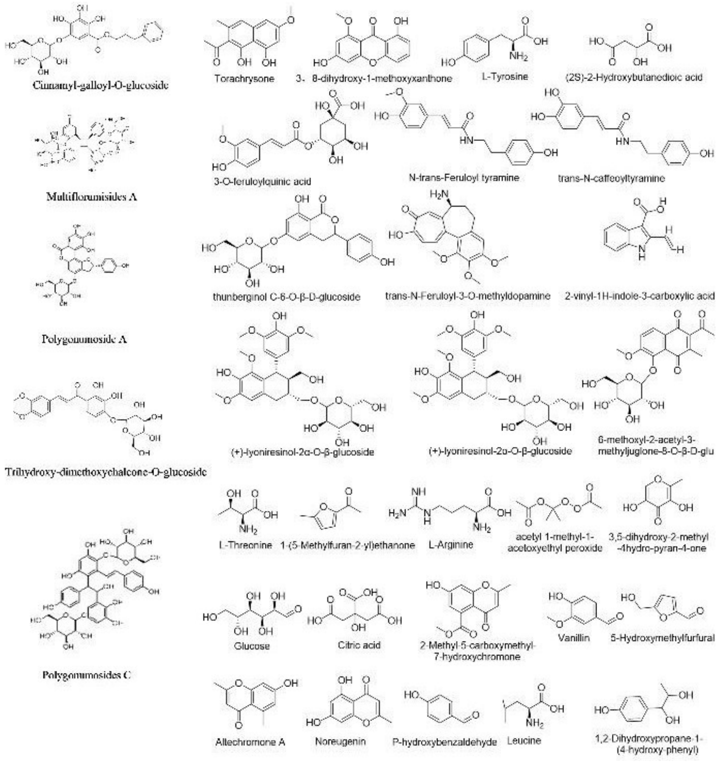

Figure S1. Chemical structures of compounds identified in PMRP. Glc: glucoside; Mal: malonyl; Ace: acetyl; EA: Emodin anthrone; Gal: galloyl; Cou: coumaroyl; Fer:feruloyl; Caf: caffeoyl; Rha: rhamnose; Xyl: xylose; Gly: glycerol.
